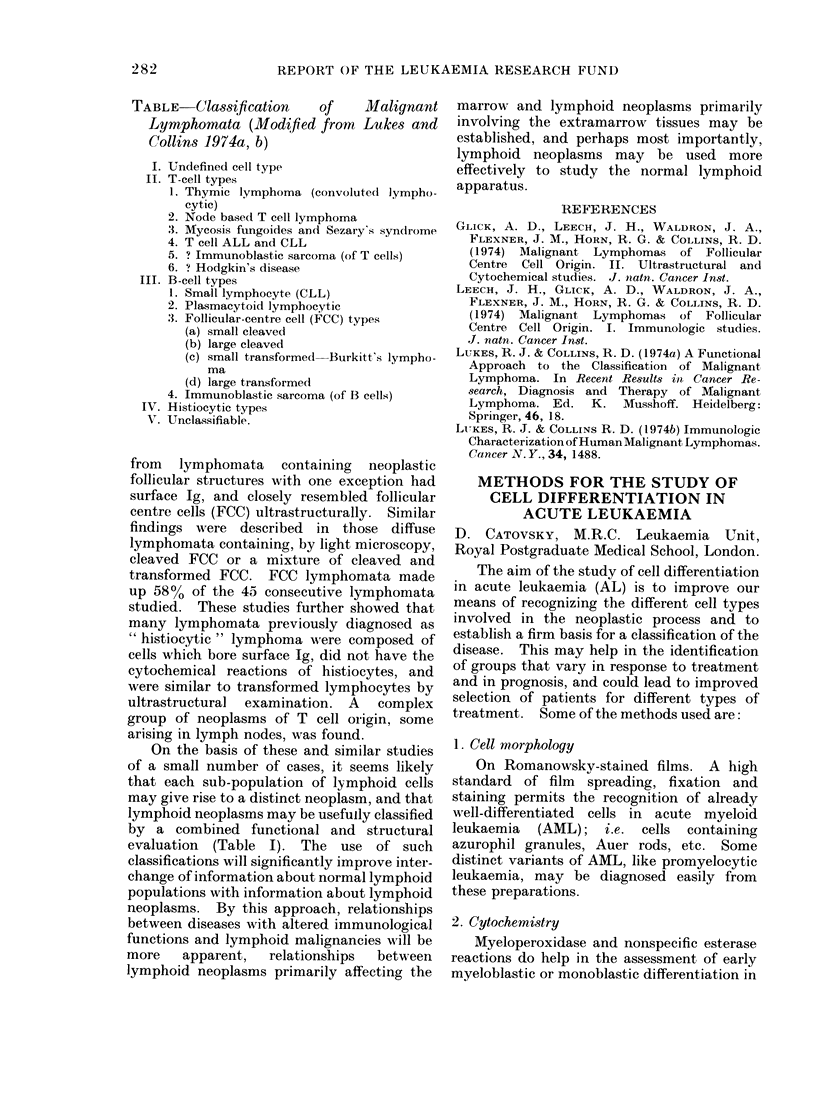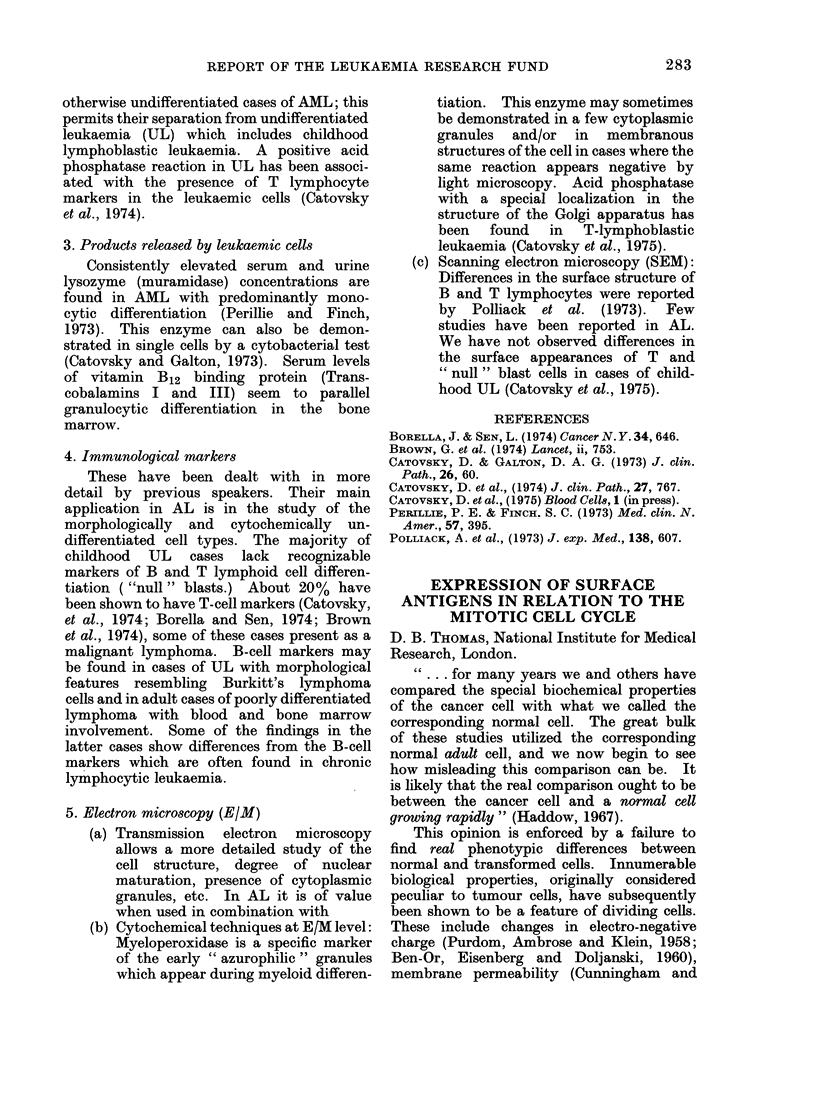# Proceedings: Methods for the study and cell differentiation in acute leukaemia.

**DOI:** 10.1038/bjc.1975.220

**Published:** 1975-08

**Authors:** D. Catovsky


### METHODS FOR THE STUDY OF CELL DIFFERENTIATION IN ACUTE LEUKAEMIA

D. CATOVSKY, M.R.C. Leukaemia Unit, Royal Postgraduate Medical School, London.

The aim of the study of cell differentiation in acute leukaemia (AL) is to improve our means of recognizing the different cell types involved in the neoplastic process and to establish a firm basis for a classification of the disease. This may help in the identification of groups that vary in response to treatment and in prognosis, and could lead to improved selection of patients for different types of treatment. Some of the methods used are:

### 1. *Cell morphology*

On Romanowsky-stained films. A high standard of film spreading, fixation and staining permits the recognition of already well-differentiated cells in acute myeloid leukaemia (AML); *i.e.* cells containing azurophil granules, Auer rods, etc. Some distinct variants of AML, like promyelocytic leukaemia, may be diagnosed easily from these preparations.

### 2. *Cytochemistry*

Myeloperoxidase and nonspecific esterase reactions do help in the assessment of early myeloblastic or monoblastic differentiation in otherwise undifferentiated cases of AML; this permits their separation from undifferentiated leukaemia (UL) which includes childhood lymphoblastic leukaemia. A positive acid phosphatase reaction in UL has been associated with the presence of T lymphocyte markers in the leukaemic cells (Catovsky *et al.*, 1974).

3. *Products released by leukaemic cells*

Consistently elevated serum and urine lysozyme (muramidase) concentrations are found in AML with predominantly monocytic differentiation (Perillie and Finch, 1973). This enzyme can also be demonstrated in single cells by a cytobacterial test (Catovsky and Galton, 1973). Serum levels of vitamin $B_{12}$ binding protein (Transcobalamins I and III) seem to parallel granulocytic differentiation in the bone marrow.

4. *Immunological markers*

These have been dealt with in more detail by previous speakers. Their main application in AL is in the study of the morphologically and cytochemically undifferentiated cell types. The majority of childhood UL cases lack recognizable markers of B and T lymphoid cell differentiation ("null" blasts.) About 20% have been shown to have T-cell markers (Catovsky, *et al.*, 1974; Borella and Sen, 1974; Brown *et al.*, 1974), some of these cases present as a malignant lymphoma. B-cell markers may be found in cases of UL with morphological features resembling Burkitt's lymphoma cells and in adult cases of poorly differentiated lymphoma with blood and bone marrow involvement. Some of the findings in the latter cases show differences from the B-cell markers which are often found in chronic lymphocytic leukaemia.

5. *Electron microscopy (E/M)*

(a) Transmission electron microscopy allows a more detailed study of the cell structure, degree of nuclear maturation, presence of cytoplasmic granules, etc. In AL it is of value when used in combination with
(b) Cytochemical techniques at E/M level: Myeloperoxidase is a specific marker of the early "azurophilic" granules which appear during myeloid differentiation. This enzyme may sometimes be demonstrated in a few cytoplasmic granules and/or in membranous structures of the cell in cases where the same reaction appears negative by light microscopy. Acid phosphatase with a special localization in the structure of the Golgi apparatus has been found in T-lymphoblastic leukaemia (Catovsky *et al.*, 1975).
(c) Scanning electron microscopy (SEM): Differences in the surface structure of B and T lymphocytes were reported by Polliack *et al.* (1973). Few studies have been reported in AL. We have not observed differences in the surface appearances of T and "null" blast cells in cases of childhood UL (Catovsky *et al.*, 1975).

REFERENCES

Borella, J. & Sen, L. (1974) *Cancer N.Y.* **34**, 646.
Brown, G. *et al.* (1974) *Lancet*, ii, 753.
Catovsky, D. & Galton, D. A. G. (1973) *J. clin. Path.*, **26**, 60.
Catovsky, D. *et al.*, (1974) *J. clin. Path.*, **27**, 767.
Catovsky, D. *et al.*, (1975) *Blood Cells*, **1** (in press).
Perillie, P. E. & Finch. S. C. (1973) *Med. clin. N. Amer.*, **57**, 395.
Polliack, A. *et al.*, (1973) *J. exp. Med.*, **138**, 607.